# Novel severe acute respiratory syndrome coronavirus 2 (SARS-CoV-2) co-infection with HIV: clinical case series analysis in North Central Nigeria

**DOI:** 10.11604/pamj.2020.37.47.24200

**Published:** 2020-09-10

**Authors:** Adamu Ishaku Akyala, Chinwe Juliana Iwu

**Affiliations:** 1 Department of Microbiology, Faculty of Natural and Applied Sciences, Nasarawa State University, Keffi, Nasarawa State, Nigeria,; 2 Department of Nursing and Midwifery, Faculty of Medicine and Health Sciences, Stellenbosch University, Cape Town, South Africa

**Keywords:** Coronavirus disease 2019 (COVID-19), transmission, clinical characteristics, viral shedding, HIV and COVID-19 co-infection, Nigeria

## Abstract

SARS-CoV-2 has created a global public health emergency with significant mortality and morbidity for people living with HIV (PLWH). Preliminary data reveals persons with immune-compromised status are at risk of developing adverse clinical outcomes from SARS-CoV-2. We aimed to characterise clinical outcomes of HIV patients co-infected with SARS-CoV-2 infection in Nasarawa State, North Central Nigeria. We followed four (4) hospitalised HIV patients that tested positive to SARS-CoV-2 in Nasarawa State and characterised their laboratory findings and clinical outcomes. The consent of the cases was sought and they agreed that their clinical data be published. Real-time reverse transcriptase polymerase chain reaction (RT-PCR) tests for SARS-CoV-2 nucleic acid were performed using nasopharyngeal swabs (novel coronavirus PCR fluorescence diagnostic kit, BioGerm medical biotechnology) at the Nigeria Centre for Disease Control (NCDC) in Abuja, Nigeria. Our study reveals mild clinical outcome among HIV patients with SARS-CoV-2 co-infection. There is need for a syndemic framework to be used to conceptualise SARS-CoV-2 impact among HIV patients and an urgent need to strengthen healthcare programmes within Nigeria.

## Introduction

An epidemic of respiratory disease caused by severe acute respiratory syndrome coronavirus 2 (SARS-CoV-2) began in Wuhan, China and has spread to other countries of the world [1]. In the past 25 years, several highly transmissible respiratory viruses with epidemic potential have emerged. The most significant pandemics of the 20^th^ century, notably, were influenza viruses in 1918, 1957 and 1968. Since then, several other viral respiratory pathogens have emerged including Middle East respiratory syndrome coronavirus (MERS-CoV), adenovirus-14 and virulent strains of influenza viruses. Soon after the discovery of SARS, new coronaviruses including NL63 and HKU1 were identified [2, 3]. The rapid emergence of these respiratory viruses underscores their epidemic potential and overall threat to global health security. In 2020, a global alert was issued for an emerging yet unknown illness known as severe acute respiratory syndrome (SARS) caused by a novel SARS-CoV-2 [2]. According to the World health Organization (WHO), this novel SARS-CoV-2 virus outbreak has, as of 2: 33pm CEST, 29^th^ May 2020, resulted in 5,701,337 confirmed cases and 357,688 deaths, [3] with substantial economic impact. Africa has as at same date, recorded 92,929 cases and 2,427 deaths [3]. The USA Center for Disease and Control (CDC) in March, 2020 revealed that persons living with HIV will be faced with multiple morbidities which might heighten the risk of severe health outcome from the SARS-CoV-2 [4]. Worse clinical outcomes from other associated commodities like; cardiovascular diseases, hypertension, diabetes and other underlying respiratory tract diseases and cancer among others [5]. About 37.9 million people are infected with HIV and AIDS globally [6] and there is paucity of information regarding the impact of SARS-CoV-2 on this population in Nigeria. This study was therefore aimed at understanding clinical outcome of cases of PLHIV who are infected by the in Nasarawa State, North Central Nigeria.

## Methods

We performed a case series observational study among SARS-CoV-2 patients who had HIV as an underlying disease between 29^th^ March 2020 to 25^th^ May 2020. Patients who tested positive to SARS-CoV-2 but are not HIV positive where excluded from the study. We collated demographic data, history, hospital admissions and laboratory results such as CD4 count and HIV treatment regimens.

## Results

All the four cases were female, sex workers who were intercepted on their way to Nasarawa State from a high-risk state (province), Kano, Nigeria. They were treated with proteases and intergrase inhibitor based antiretroviral therapy (ART), with CD4+ count above 200 cells per uL. We conducted a nasopharyngeal and throat swab for SARS-CoV-2, followed by a RT-PCR assay and the result returned positive. They were admitted after they tested positive in the isolation and treatment center and subsequently were managed for SARS-Cov-2. They had other underlying comorbidities which include: diabetes, chronic sinusitis, pulmonary tuberculosis with accompanying signs and symptoms of COVID-19 including malaise, cough, fever and headaches, and decrease in lymphocyte and white blood cell count. On admission at the two centers in Nasarawa State, their average body temperature, oxygen saturation and respiratory rates were 39°C, 80% and 30 breaths per minute respectively. These cases received norfloxacin 400mg once daily for five days, γ-globulin 400mg/kg once daily for three days and methylprednisolone 0.8mg/kg once daily for three days through intravenous route. The cases are in stable condition and were discharged from the treatment and isolation center. They were asked to self-isolate for two weeks in their homes. Table 1 shows the baseline HIV status, clinical outcomes and demographic characteristics of four cases of novel SARS-CoV-2 co-infection with HIV.

**Table 1:** baseline HIV status, clinical outcomes and demographic characteristics of novel SARS-CoV-2 co-infection with HIV among cases in Nasarawa State

Characteristics	Case 1	Case 2	Case 3	Case 4
Age (years)	25	30	23	40
Gender	Female	Female	Female	Female
Comorbidities	Diabetic	Asthmatic	Chronic sinusitis	Tuberculosis
HIV-risk factor and exposure	Sex worker	Sex worker	Sex worker	Sex worker
Year of HIV diagnosis	2017	2010	2020	2015
Last CD4 cell count (cells per μL)	260	302	251	205
Last CD4:CD8 ratio	1.3	2.1	0.6	0.11
HIV viral load at or before admission (copies per mL)	>50	600,000	12,650	30,030
ART-regimen before admission	Abacavir, lamuvudine	Tenofovir, emtricitabine	Alafenamide, tenofovir	Abacavir, lamivudine
Symptoms	Cough, malaise	Headache	Cough	Malaise, Cough
Temperature	Fever (36.5°C)	Fever (36.8°C)	Fever (38.9°C)	Fever (39.9°C)
Diagnosis	Lower respiratory tract infection	Lower respiratory tract infection	Lower respiratory tract infection	Lower respiratory tract infection
Duration of onset symptoms (days)	3	2	5	4
**Laboratory results**				
Lymphocyte (cells per 106/L) F	9001	7822	6232	4601
White blood cell count (cells per 106/L)	6420	6509	7456	9801
Severity of infection	Mild	Mild	Mild	Mild

## Discussion

Antiretroviral therapy (ART) has been shown to increase the lifespan of people living with HIV and AIDS in sub-Saharan Africa where millions of people are living with HIV. Although clinical complications arising from long-term ART use and aging have been revealed [4, 7], comorbidities such as chronic lung disease, hypertension and diabetes, are associated risk factors within this population. These comorbidities have also been identified as the risk factors for severe SARS-CoV-2 disease [8] which is a cause for concern. On the other hand, severe complications from SARS-CoV-2 might be seen in younger likewise older people living with HIV and AIDS. Such risk is predicated on the fact that PLWH under age 50 are both less likely to be diagnosed (and in effect more likely to be immunocompromised) and also less likely to access and be retained in care, yielding viral suppression of a mere 37% on those age 25-34 [8]. The clinical outcome of patients with HIV and SARS-CoV-2 co-infection in this study suggests that antiretroviral drugs may have protective effects on PLWIH as they were stable upon discharge, despite the comorbidities they presented with.

## Conclusion

Our study therefore, reveals significant mild clinical outcome among HIV patients with SARS-CoV-2 co-infection. There is need for a syndemic framework to be used to conceptualise SARS-CoV-2 impact among HIV patients and an urgent need to strengthen healthcare programmes within Nigeria. Furthermore, there is need for health system and programme strengthening as SARS-CoV-2 may be an opportunistic infection among PLWH. There is need to have a future contingency plan in addressing HIV-SARS-CoV-2 co-infection by providing structural drivers for disease surveillance. Understanding viral interaction is key in effective treatment of HIV-SARS-CoV-2 co-infection. Worthy of note, antiretroviral treatments in form of pre-exposure prophylaxis (PrEP) or antiretroviral therapy ART for PLWH have some predictive protection against SARS-CoV-2. Our study reveals that HIV-positive patients may not experience significantly worse outcomes in SARS-CoV-2 infection this is because of their antiretroviral regimen which may provide them with some protection.

### What is known about this topic

- Antiretroviral therapy (ART) has been shown to increase the lifespan of people living with HIV and AIDS in sub-Saharan Africa where millions of people are living with HIV (PLWH);
- Comorbidities such as chronic lung disease, hypertension and diabetes, are associated risk factors among PLWH;
- Similar comorbidities are also associated with risk of severe outcomes with people infected with SARS-CoV-2.

### What this study adds

- The course of COVID-19 in PLWH who are on ART seems to be mild.
